# *Fusarium Oxysporum* f. sp. *Cannabis* Isolated from *Cannabis Sativa* L.: In Vitro and In Planta Biocontrol by a Plant Growth Promoting-Bacteria Consortium

**DOI:** 10.3390/plants10112436

**Published:** 2021-11-11

**Authors:** Marika Pellegrini, Claudia Ercole, Carmelo Gianchino, Matteo Bernardi, Loretta Pace, Maddalena Del Gallo

**Affiliations:** Department of Life, Health and Environmental Sciences, University of L’Aquila, Coppito 1, 67100 L’Aquila, Italy; claudia.ercole@univaq.it (C.E.); carmelo.gianchino@graduate.univaq.it (C.G.); matteo.bernardi1@graduate.univaq.it (M.B.); loretta.pace@univaq.it (L.P.); maddalena.delgallo@univaq.it (M.D.G.)

**Keywords:** PGPB, biocontrol, *Fusarium* wilt, industrial hemp, ITS barcoding, scanning electron microscopy

## Abstract

Industrial hemp (*Cannabis sativa* L.) is a multipurpose plant used in several fields. Several phytopathogens attack hemp crops. *Fusarium oxysporum* is a common fungal pathogen that causes wilt disease in nurseries and in field cultivation and causes high losses. In the present study, a pathogenic strain belonging to *F. oxysporum* f. sp. *cannabis* was isolated from a plant showing *Fusarium* wilt. After isolation, identification was conducted based on morphological and molecular characterizations and pathogenicity tests. Selected plant growth-promoting bacteria with interesting biocontrol properties—*Azospirillum brasilense*, *Gluconacetobacter diazotrophicus*, *Herbaspirillum seropedicae* and *Burkholderia ambifaria*—were tested against this pathogen. In vitro antagonistic activity was determined by the dual culture method. Effective strains (in vitro inhibition > of 50%) *G. diazotrophicus*, *H. seropedicae* and *B. ambifaria* were combined in a consortium and screened for in planta antagonistic activity in pre-emergence (before germination) and post-emergence (after germination). The consortium counteracted *Fusarium* infection both in pre-emergence and post-emergence. Our preliminary results show that the selected consortium could be further investigated as an effective biocontrol agent for the management of this pathogen.

## 1. Introduction

Hemp (*Cannabis sativa* L.) is a crop with a rich and ancient history and is grown all over the world. Its widespread cultivation is because of the versatility of this plant in a variety of fields. Hemp can be used in the textile and manufacturing industries and for the production of biobased materials [1]. The metabolites of hemp (e.g., cannabinoids, phenolic compounds, vitamins and proteins) can be used in pharmaceutical, nutraceutical and food industries [2,3,4]. Hemp crops are threatened by attacks from viruses, bacteria and fungi that penetrate through the surfaces of leaves, stems and roots; spread within the tissues; and colonize the entire plant [5]. Some of these plant pathogens can cause significant damages to hemp plants by blocking plant development and causing metabolic disorders, leaves shriveling or roots destruction [6]. Previously reported pathogens that can cause wilting and collapse of *C. sativa* plants include *Fusarium oxysporum* f. sp. *cannabis* (FOC) and *F. oxysporum* f. sp. *vasinfectum* (FOV) [7]. FOV *forma specialis* affects a wide range of hosts, while FOC is specific to hemp and can result in complete crop loss. Symptoms of FOC pathogenesis begin with dark spots on lower leaflets, rapid wilting of leaves, covering of stem cortex with mycelium and death of the plant [8]. It is possible to use naturally occurring plant-microbe interactions to counteract the attacks of phytopathogens. Through various direct and indirect mechanisms, Plant Growth-Promoting Bacteria (PGPB) can be used as sustainable biocontrol agents against many phytopathogens [9,10]. In the literature, biocontrol of hemp *Fusarium* has been described for *Burkholderia cepacia, Pseudomonas fluorescens* and *Streptomyces griseoviridis* and the beneficial fungi *Trichoderma lignorum* and *Glomus intraradices* [7]. This scarcity of biocontrol agents requires the search for new effective biocontrol agents. Our study aims to investigate the efficacy of a bacterial consortium for the control of hemp *Fusarium*. Among the PGPB belonging to our Environmental Microbiology laboratory collection, we selected *Azospirillum brasilense, Gluconacetobacter diazotrophicus, Burkholderia ambifaria* and *Herbaspirillum seropedicae*, provided by several colleagues (Y. Okon, J. Döbereiner and T. Heulin). Since these bacteria have shown in planta biocontrol against other *F. oxysporum* f. sp. *radicis-lycopersici* [11] and good biostimulatory abilities on *C. sativa* ‘Finola’ [12], we hypothesized that they could be an effective biocontrol agent for hemp against fusariosis. These bacterial species live in association with many crops, are associated with plant roots and promote plant growth through various direct (e.g., hormone production) and direct mechanisms (e.g., production of biocontrol molecules) [13,14,15,16].

We isolated a FOC *forma specialis* from a plant with specific symptoms (i.e., wilted leaves with yellow-tan colour and cortex covered by fungal mycelium). This FOC strain was characterized by internal transcribed spacer (ITS) sequencing and by pathogenesis assay. The antagonistic activities of the individual strains and the effective strain’s PGPB consortium (*B. ambifaria*, *G. diazotrophicus* and *H. seropedicae*) against the FOC pathogen were first evaluated in vitro, examining the inhibitory ability by dual culture method and the morphological changes of the mycelium in the presence of PGPB by scanning electron microscopy (SEM). The PGPB consortium of effective strains was tested in planta in order to verify the induced protection under pre-emergence (infection before germination) and post-emergence (infection after germination) conditions.

## 2. Results

### 2.1. Fungal Isolate Morphological and Molecular Identification

**Figure 1:**
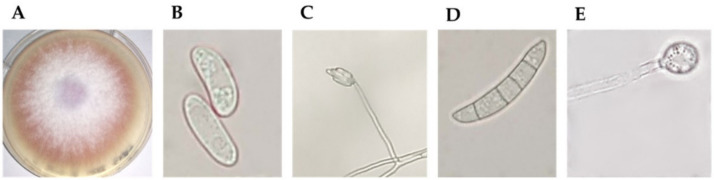
Putative *Fusarium oxysporum* morphological characteristics. In the figure: (**A**) pale violet colour of mycelia; (**B**) oval-shaped microconidia; (**C**) false heads of microconidia on a monophialide; (**D**) sickle-shaped macroconidia; (**E**) terminal chlamydospore.

*Fusarium* isolates obtained on Selective *Fusarium* Agar (SFA) [17] were screened based on macroscopic and microscopic observations. Based on the colour of mycelium and growth rate on Potato Dextrose Agar (PDA, Oxoid, United Kingdom) and microconidia and macroconidia on Soil Agar (SA) [17], a putative *Fusarium oxysporum* isolate was selected. As shown in Figure 1, the 8 cm mycelium that developed from the isolate after 7 days has a pale purple/deep pink colour (Figure 1A). Oval-shaped microconidia (Figure 1B) are formed in false heads on monophialides (Figure 1C); the macroconidium has five septa (Figure 1D), and single and terminal chlamydospores are present (Figure 1E).

**Figure 2:**
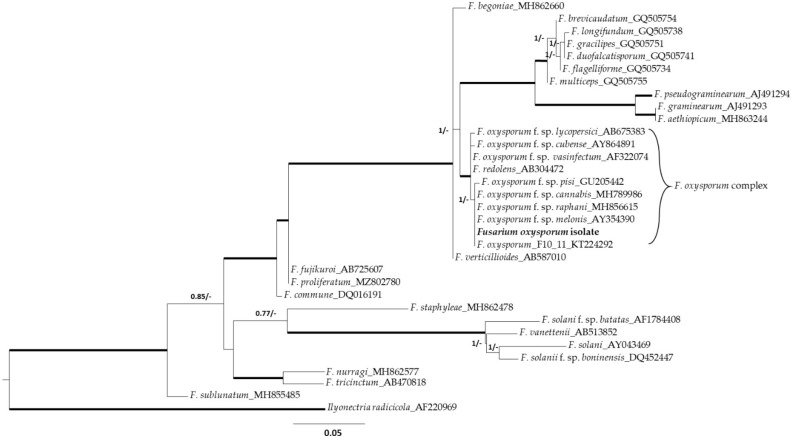
Phylogenetic tree inferred from maximum likelihood and Bayesian analyses from internal transcribed spacer (ITS) regions of 24 representative species of *Fusarium*, the isolate of this study and the *Ilyonectria radicicola* outgroup. Thickened branches indicate those that are supported both by likelihood bootstrap values of >70% and by Bayesian posterior probabilities of >95%. The definition of MrBayes and RAxML percentages bootstraps are defined next to the branches at each node (probabilities/bootstrap). Scale bar represents the number of substitutions per nucleotide site for a unit of branch length.

The isolate was then characterized by ITS sequencing and identified with 100% identity as *Fusarium oxysporum* (Figure 2). Phylogenetic analyses grouped the FOC isolate with a high degree of sequence identity (99–100%) within the *Fusarium oxysporum* complex. Figure 2 shows the phylogenetic tree inferred from maximum likelihood and Bayesian analyses from ITS regions of 24 representative species of *Fusarium*, the isolate of this study and the *Ilyonectria radicicola* outgroup. The *formae speciales* that caused pathogenicity on hemp include *F. oxysporum* f. sp. *vasinfectum*, which attacks other plants such as *Capsicum annuum* and *Medicago sativa*, and *F. oxysporum* f. sp. *cannabis*, which occurs only on hemp [18]. We infected the seeds of *C. sativa*, *M. sativa* and *C. annuum* at sowing with a spore solution of 10^6^ CFU mL^−1^ and observed the development of pathogenesis for 20 days. The seeds of *C. sativa* that germinate developed rachitic plants with dark spots on the leaves and wilting of the leaves. In *M. sativa* and *C. annuum*, there were no changes in seed germination, plant development (no wilting leaves) and morphology (no black spots). Therefore, based on the ability to induce pathogenesis on *C. sativa* and not on *Medicago sativa* and *C. annuum*, the isolate was classified as *F. oxysporum* f. sp. *cannabis*. 

### 2.2. In Vitro Antagonistic Activity

**Figure 3:**
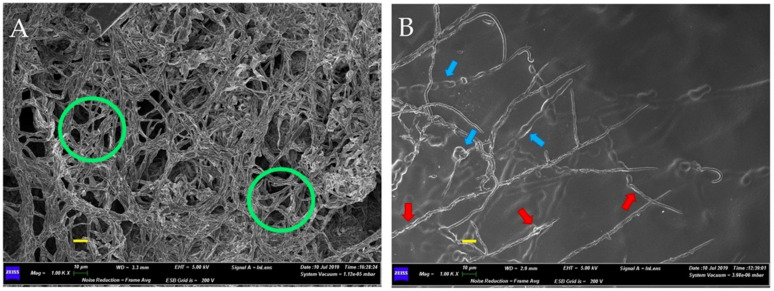
Scanning Electron Microscope (SEM) micrographs at 1000X showing differences in mycelium development of *Fusarium oxysporum* f. sp. *cannabis*. (**A**) Control mycelium with continuous and normal hyphae and branching; (**B**) mycelium with swelling and vacuolation of the hyphae present in an interaction zone between *F. oxysporum* f. sp. *cannabis* and the bacterial consortium formed by *Gluconacetobacter diazotrophicus*, *Herbaspirillum seropedicae* and *Burkholderia ambifaria*. Scale bars (in yellow) 10 µm.

PGPB antagonistic activity against FOC was tested in vitro by dual culture (cultivation of single bacteria/consortium and FOC on PDA medium). Effective growth inhibition was assumed when the percentage of inhibition was higher than 20%. Based on the distribution of mycelium in the centre and bacterial streaks at the edges of the plate, values below 20% were associated with the growth of mycelium on and across bacterial streaks. The percentages of inhibition obtained after 7 days of culture are presented in Table 1. The in vitro antagonistic activity of *B. ambifaria*, *G. diazotrophicus* and *H. seropedicae* was statistically similar (*p* > 0.05), with an average inhibition of 68%. For these bacteria, mycelial growth ceased before the bacterial streaks (Figure 3A). For *A. brasilense*, no effective inhibition was observed, the inhibition was less than 20% and mycelium grew across the bacteria streaks (Figure 3B). The latter was excluded from the consortium, which comprised equal amounts of *B. ambifaria*, *G. diazotrophicus* and *H. seropedicae* broth cultures. The combination of strains in the consortium did not alter antagonistic activity (no statistically significant differences from the values of the individual strains, *p* > 0.05), with an inhibition rate of 71%.

### 2.3. Bacterial Effects on Fungal Mycelium

**Figure 4:**
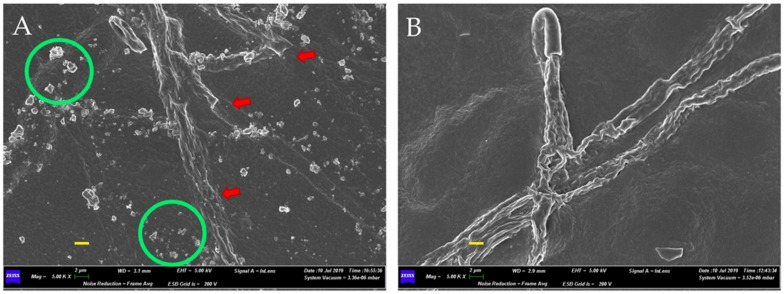
Scanning Electron Microscope (SEM) micrographs at 5000 X that show the abnormalities of the *Fusarium oxysporum* f. sp. *cannabis* mycelium. In the presence of the bacterial consortium formed by *Gluconacetobacter diazotrophicus*, *Herbaspirillum seropedicae* and *Burkholderia ambifaria*, the mycelium presented irregular and desegregated hyphae (**A**), with a distorted development (**B**). Scale bars (in yellow) 2 µm.

Scanning electron microscopy (SEM) observations of the inhibition zones of the consortium–FOC dual cultures showed the effects of the bacterial consortium on the fungal mycelium. Figure 3 and Figure 4 present the micrographs obtained by SEM. In the absence of PGPB during growth (Figure 3A), the mycelium exhibits normal growth with continuous overlapping and abundant hyphae (green circles). In the presence of PGPB (Figure 3B), the mycelium is discontinuous, with sparse and deformed hyphae (swelling and vacuolation are shown by blue and red arrows, respectively). The 5000× micrograph details in Figure 4 show the bacterial effects on the hyphal structures. Figure 4A shows the disaggregation of fungal branches (arrows) and lytic fragments (circles), while Figure 4B shows the thinning of hyphal branches.

### 2.4. In Planta Biocontrol

**Figure 5:**
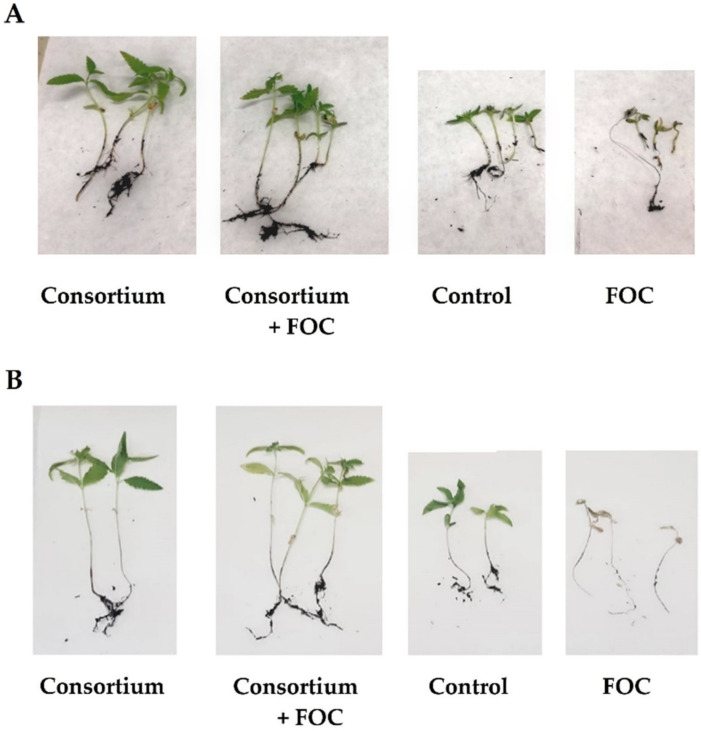
Comparison of experimental units obtained for pre-emergence (**A**) and post-emergence (**B**) experiments. In the figure: FOC, *Fusarium oxysporum* f. sp. *cannabis*.

The ability of the consortium, formed by *G. diazotrophicus*, *H. seropedicae* and *B. ambifaria*, to induce protection against FOC in *C. sativa* was investigated in pre-emergence and post-emergence pot experiments. Figure 5 shows the comparisons of the four experimental units for pre-emergence (Figure 5A) and post-emergence (Figure 5B) trials.

In both pre-emergence and post-emergence trials, treatment of the plant with the bacterial consortium alone (Consortium) promoted good plant growth. Similar plant development was observed in Consortium + FOC (presence of the bacterial consortium and the fungal pathogen). The plants under these two experimental conditions were longer than those of the control. Under the experimental condition Consortium + FOC, the plants were healthier than those of FOC (not treated with bacteria and infected with the fungal pathogen). The results of the pre-emergence and post-emergence in planta trials are shown in Table 2.

Two-way analysis of variance (ANOVA) followed by Fisher’s least significant difference (LSD) post hoc test showed that the two variables, Condition and Trial, and their interaction (Condition × Trial) had a significant effect. The best plant growth parameters were obtained under the Consortium experimental condition followed by Consortium + FOC and Control. The lowest results were registered under FOC. Except for damages, plant growth parameters of pre-emergence and post-emergence trials differed significantly. The summary of multiple pairwise comparisons for Condition x Trial (Fisher (LSD) interaction is presented in Table S1 in Supplementary Materials.

In the pre-emergence trial, FOC infection significantly reduced germination, with a decrease of −45% compared to the control. Plants that germinated and grew despite the fungal infection (FOC) exhibited damages and recorded a decrease in all growth parameters. Plant height and root length decreased significantly (*p* < 0.05) compared to control (−33% and −44%, respectively). The number of true leaves, chlorophylls content and chlorophyll a/b ratio (*p* < 0.05) was also lower than the control (*p* < 0.05). In the absence of fungal infection (Consortium), the bacteria promoted good plant growth and development, with the highest values for all parameters (*p* < 0.05). Plant growth and development promoted by the bacterial consortium was flawed in the presence of fungal infection (Consortium + FOC). However, the severity of infection was less and resulted in a lower decrease in germination (−11% than control). For the plants under Consortium + FOC, fewer damages and better growth parameters were recorded compared to those under FOC. The number of true leaves and the chlorophyll a/b ratio was statistically comparable to the control (*p* > 0.05), while plant height, root length and chlorophylls contents had higher values than the control (*p* < 0.05).

In the post-emergence experiment, the FOC infection (FOC) induced a massive loss of plants (plant survival −58%). Plants that survived fungal infection exhibited extended damages and recorded the lowest growth parameters (*p* < 0.05). In the presence of the bacterial consortium, there was a substantial reduction in fungal infection symptoms (Consortium + FOC parameters lower than Consortium, *p* < 0.05). The improvement in all parameters investigated highlighted the effective antagonistic activity of bacterial consortium against FOC (Consortium + FOC parameters higher than FOC, *p* < 0.05). The number of true leaves and chlorophylls content was similar to the control (*p* > 0.05), while plant height and root length were higher than the control (*p* < 0.05).

## 3. Discussion

In this study, the application of a consortium of three beneficial bacteria significantly reduced FOC disease in both pre-emergence and post-emergence trials. The biocontrol agents available for preventing and countering FOC are limited, and the literature lacks scientific studies on biocontrol agents against *forma specialis*. However, our findings are consistent with previous studies on microbial consortia as biocontrol agents against plant fungal diseases [19]. The biocontrol potential of the bacterial strains that form our consortium against *Fusarium* spp. has been described in various studies. Simonetti et al. demonstrated that *B. ambifaria* has strong activities against *Fusarium* spp. (i.e., *F. graminearum*, *F. oxysporum* and *F. solani*) when using fusaric acid (responsible for the disease) as an energy source [20]. *B. ambifaria* is a valid biocontrol strain thanks to a set of numerous diffusible and volatile antifungal molecules. Among the diffusible molecules, we can find the powerful antifungals burkholdines, occidiofungin, pyrrolnitrin and 4-hydroxy-2- alkylquinoline [21,22,23]. *B. ambifaria* volatile antifungal compounds include dimethyl disulfide, dimethyl trisulfide, 4-octanone, S-methyl methanethiosulphonate, 1-phenylpropan-1-one and 2-undecanone [13]. Mehnaz and Lazarovits showed in vitro inhibitory activity of *G. diazotrophicus* against *Fusarium* spp. [24]. The same results were reported by Logeshwarn against *F. oxysporum* of sweet potato, ascribing the inhibition capabilities to 2,4-diacetylphloroglucinol, pyrrolnitrin and pyoluteorin [14]. Weber et al. described effective control of *F. oxysporum* f. sp. *cubense* in banana seedlings in the presence of the co-inoculation of *H. seropedicae* and *Burkholderia cepacia* [25]. *H. seropedicae* intervenes in the modulation of the host plant’s defence responses [15,26] and produces siderophores (serobactins) that contribute to competition within the plants [27,28].

The production of metabolites by beneficial bacteria is essential to help the plant fight fungal diseases by interfering with the growth and activities of pathogens. In addition to diffusible (e.g., organic acids, lipopeptides and pyrroles) and volatiles (e.g., hydrocyanide, ammonia and sulphides), other metabolites can counteract fungal infection. Lytic enzymes, for example, can directly break down constitutive polymeric compounds (i.e., chitin, proteins and DNA) [29]. Another effect exerted by beneficial bacteria is competition for nutrient sources particularly against soil-borne pathogens, such as *Fusarium* [29]. Trophic competition can involve carbon, nitrogen and iron and can be an effective biocontrol mechanism against phytopathogenic fungi [30]. Biocontrol inoculants based on microbial consortia are an effective strategy for crop protection against phytopathogens [31]. Bacterial inoculation induces the activation of the defence response of host plants and increases nutrient uptake and root structure by reducing the propagation of pathogens [32]. The presence of more strains broadens the antagonistic spectrum and improves performance [33]. 

Other direct plant growth-promoting traits also counteracted fungal pathogens. Our findings demonstrated that the bacterial consortium enhanced the growth of plants both in pre-emergence and post-emergence trials. This positive effect on plant growth is related to the ability of *B. ambifaria*, *G. diazotrophicus* and *H. seropedicae* in producing phytohormones, solubilizing nutrients and fixing atmospheric nitrogen [34].

Fungal diseases are a major concern in agriculture given the huge losses induced annually. The control of fungal diseases in crops is achieved by using agrochemicals. These substances, extensively applied in prevention campaigns, have resulted in severe consequences for the environment and human health. Pollution of soil, groundwater and surface water by agrochemicals is toxic to both humans and animals and induces the growth of algae, which unbalances the life cycle of aquatic animals [35]. This situation drives the scientific community and agriculture to search for valid alternative techniques for the control of fungal infections. In this study, we focused our attention on hemp. Many fungal diseases threaten the crops of this multipurpose plant every year. FOC is a devastating fungal disease of hemp [7]. The severity of its pathogenesis is so strong that this fungus is used as a bioherbicide to destroy the illegal fields of *C. sativa* subsp. *indica* [36]. To the best of our knowledge, this study is the first report on the biocontrol ability of a bacterial consortium against FOC. Further studies should be directed toward the evaluation of this consortium in greenhouse (repeated experiments with different light and soil characteristics and a major number of plants) and open field experiments (different pedoclimatic conditions). In order to clarify the mechanism’s underlying the biocontrol activity, the characterization of the bioactive molecules produced by the bacteria against FOC should also be carried out, as well as the response of the plant to fungal infection in the presence of bacteria. The preliminary results obtained so far suggest that this consortium may have activity against *F. oxysporum* ff. spp. and other fungal pathogens [11]. Future research should investigate the biocontrol ability of the consortium against *F. oxysporum* f. sp. *vasinfectum* and other fungal pathogens in hemp and other crop plants. More detailed studies of the translation elongation factor alpha genetic region of the pathogenic fungus could also provide additional information on the phylogeny of the isolate [37].

## 4. Materials and Methods

### 4.1. Fungal Strain Isolation and Growth Conditions

The stem of a hemp plant with classic symptoms of *Fusarium* wilt was sampled from M.A.D. Biofarm SS field (42.0302, 13.4421, Avezzano, Italy) in August 2018. Several pieces of the stem cortex (~3 × 3 cm) with a clear cover of mycelium (15–20 pieces) were sampled with sterile blades, placed in sterile plastic bags and transferred to the laboratory. Small pieces of cortex tissue (0.5–1 cm) were treated with a 0.5% sodium hypochlorite solution for 30 s, 70% ethanol solution for 20 s and rinsed five times in sterile distilled water. Pieces were left to dry under hood flow and plated on SFA, supplemented after autoclaving with 20 mL L^−1^ of 5% streptomycin stock solution (Sigma-Aldrich, St. Louis, MO, USA), 12 mL L^−1^ of 1% neomycin stock solution (Sigma-Aldrich, St. Louis, MO, USA) and 13 mL L^−1^ of 0.5% 2,6-dichloro-4-nitroanaline ethanol stock solution (Sigma-Aldrich, St. Louis, MO, USA) [17]. SFA plates were incubated at 25 °C for 5–10 days. By using a stereomicroscope placed under Gelaire TC48 laminar flow hood (class 2 cabinet (Gelaire, Sydney, Australia)) and sterile needles, single spore isolation was carried out. Emerging colonies were transferred to fresh medium and permitted to grow. The isolates were selected based on macroscopic and microscopic observations (mycelium colour and growth rate on PDA and microconidia, macroconidia on SA [17]). FOC liquid cultures were grown in 250 mL Erlenmeyer flasks containing 150 mL of Potato Dextrose Broth (PDB) at 25 °C under constant shaking (150 rpm) for 7 days (mycelial mat growth). Spore solutions were prepared from 7 days PDB cultures by filtering the broth through 4 layers of muslin cloth, centrifuging at 6000× *g* for 10 min and adjusting the density to 10^6^ by a Burker chamber [38].

### 4.2. Fungal Strain Molecular Identification

The putative *Fusarium oxysporum* isolate was identified at the species level by ITS rDNA sequencing. The primers of ITS1F-ITS4 (ITS1-F 5′-CTTGGTCATTTAGAGGAAGTAA-3′ and ITS4 5′-TCCTCCGCTTATTGATATGC-3′) [39,40] were used in the following reaction mixture: ~150 mg of fresh mycelium; 2 μL of 20 mg μL^−1^ bovine sieroalbumin solution; 1.5 μL of 5U μL^−1^ Taq polymerase solution; 5 μL Buffer 10×; 1 μL of 10 mM dNTP; 4 μL of 50 Mm MgCl_2_ solution; 2 μL ITS1F Primer forward; 2 μL ITS4 primer reverse; and sterile distilled water up to 50 μL. Negative (water) and positive (known strain) controls were included. PCR reactions were carried out in a thermal cycler (SimpliAmp™ Thermal Cycler—Applied Biosystems) with the following program: 1 cycle of 8 min at 95 °C and 30 s at 94 °C; 30 cycles lasting 30 s at 55 °C and 45 s at 728 °C; and 1 cycle from 7 min to 72 °C and re-establishment and final maintenance at a temperature of 4 °C. Sequencing was carried out by the Microsynth AG company (Balgach, Switzerland), starting from the solution of amplicons obtained by PCR checked on 1.5% agarose gel. The ITS sequences were compared with those available in the NCBI (National Center for Biotechnology Information; http://www.ncbi.nlm.nih.gov/; accessed on 2 August 2021) genetic database by using the Basic Local Alignment Search Tool (BLAST) algorithm and using only sequence identity values above 99%.

### 4.3. Phylogenetic Analysis

The phylogeny was inferred using Bayesian and Maximum Likelihood methods. *Ilyonectria radicicola* (Gerlach & Nilsson) Chaverri & Salgado (AF220969) was used as the outgroup. Bayesian search and model selection were carried out in a JModel Test [41]. We selected the best model of nucleotide substitution under the corrected Akaike’s Information Criterion. The optimal model for the rDNA region was GTR + G using MrBayes 3.2.7 [42]. Maximum Likelhood bootstrap analyses were assessed with RAxML [43] by bootstrap replicating the data matrix 1000 times in order to assess clade support. The obtained phylogenetic trees were visualised and edited by using FigTree v.1.3.1 (available at http://tree.bio.ed.ac.uk/software/figtree/; accessed on 28 October 2021). The congruence between phylogenies resulting from these two methods was determined based on sharing highly supported nodes (>70%—maximum likelihood; >95%—posterior probability).

### 4.4. Fungal Strain Formae Specialis Identification

Once the species was assigned to the isolate, the *forma specialis* was identified by utilizing a pathogenicity test. The *formae speciales* that attacked hemp included *F. oxysporum* f. sp *cannabis* (pathogen exclusive to hemp) and *F. oxysporum* f. sp. *vasinfectum* (pathogen of many plants) [18]. The pathogenicity test was carried out on *Cannabis sativa* ‘Finola,’ *Capsicum annuum* and *Medicago sativa* by using a 10^6^ CFU mL^−1^ spore solution (see Section 4.1) at sowing as a dipping solution for 20 min and by observing the development of the pathogenesis for 20 days. Seed germination rates, plant development (presence of leaf wilting) and morphology (presence of black spots) were monitored as disease symptoms.

### 4.5. Bacterial Strains and Growth Conditions

Bacterial strains *A. brasilense* ATCC 29710, *B. ambifaria* PHP7, *G. diazotrophicus* ATCC 49037 and *H. seropedicae* ATCC 35892 were cultivated in 1 L Erlenmeyer flasks containing 500 mL of T4 medium (KH_2_PO_4_ 10.99 g L^−1^; K_2_HPO_4_ 3.34 g L^−1^; Oxoid™ Yeast Extract Powder 0.05 g L^−1^; fructose 10.99 g L^−1^; 100 mL of 10× salt solution (MgSO_4_ * 7H_2_O 2 g L^−1^; NaCl 1 g L^−1^; CaCl_2_ * 2H_2_O 0.26 g L^−1^; Na_2_MoO_4_ * 2H_2_O 0.01 g L^−1^; MnSO_4_ * H_2_O 0.02; NH_4_Cl 10 g L^−1^; 2 mL of Fe-EDTA solution in 1.4% KOH; pH 6.4)) [44]. Broth cultures were grown at 30°C under constant shaking (150 rpm) for 24 h (except for *G. diazotrophicus*, cultured for 48 h).

### 4.6. In Vitro Biocontrol Activity

In vitro antagonistic activity was assessed by co-cultivation of bacterial single strains/consortium with FOC. An amount of 10 µL of 10^6^ CFU mL ^−1^ of bacterial broth cultures at the log phase (determined spectrophotometrically by comparing obtained 600 nm optical densities with growth curves) was plated with a loop forming two vertical lines at the edges of the plate and 2.5 cm away from the centre on PDA dishes Ø 90 mm dishes with 22 mL of medium; 2 lines per plate). After incubation at 28 °C (48 h for *G. diazotrophicus* and 24 h for the other bacteria), a plug (Ø 5 mm) of young FOC mycelium facing the agar (5 days old) was transferred to the centre of the dish. PDA dishes were incubated at 28 °C until the control fungal mycelium (without bacterial presence) completely covered the dish (10 days). Each trial was repeated 3 times (three independent experiments). In the presence of bacterial inhibition, we obtained no circular growth. For this reason, the growth of the fungus was measured from the centre toward both sides of bacterial streaks. The inhibition percentages were calculated as follows.

$$I\;\%=\frac{\left(\mathrm{mm}\;\mathrm{growth}\;\mathrm{control}-\;\mathrm{mm}\;\mathrm{growth}\;\mathrm{dual}\;\mathrm{culture}\right)}{\mathrm{mm}\;\mathrm{growth}\;\mathrm{control}}\;\times \;100$$

After the determination of the inhibition percentages of the individual bacteria, the consortium, formed by equal amounts of the most active bacteria (*B. ambifaria*, *G. diazotrophicus* and *H. seropedicae*) broth cultures at the log phase, was plated after vortexing, and inhibition percentages were evaluated as described above.

### 4.7. Bacterial Consortium–Pathogen Interaction

The interaction between the bacterial consortium and the FOC in in vitro biocontrol dishes was investigated by scanning electron microscopy. The part of the mycelium that develops towards the bacterial streak was sampled with the head of 1000 µL sterile pipette tips with the aid of a Greenough stereo microscope, Leica S8 APO with 8:1 apochromatic zoom. The samples were fixed overnight with a 2.5% glutaraldehyde solution in 0.05 M phosphate buffer (pH 7.3), washed with distilled water and dehydrated with a few drops of hexamethyldisilazane (HMDS—Sigma-Aldrich, St. Louis, MO, USA). The dried samples were fixed with carbon tape (Agar Scientific, Stansted, UK) on stubs and coated with chromium for SEM observations (Gemini SEM 500 SEM—Zeiss, Oberkochen, Germany). Acquisitions were performed with an acceleration voltage of 5 kV and type II secondary electrons (SE2 signal).

### 4.8. In Planta Biocontrol Activity

The in planta biocontrol activity of the consortium against FOC was assessed both during pre-emergence and post-emergence (before and after germination, respectively). The experiments were carried out by utilizing certified *Cannabis sativa* ‘Finola’ seeds (Hemp Farm Italia, Tortoreto, Italy). 

In the pre-emergence experiment, the bacterial inoculation was obtained by soaking the seeds for 20 min under constant stirring in the consortium solution (10^10^ CFU mL^−1^, determined spectrophotometrically by comparing obtained 600 nm optical densities with growth curves) and prepared with equal amounts of *B. ambifaria*, *G. diazotrophicus* and *H. seropedicae* broth cultures. After drying overnight, the bacterial density of the seeds (10^6^ CFU g^−1^) was estimated by plating serial dilutions. One gram of seed was homogenized in sterile saline with 0.1% of Tween 20 (Sigma-Aldrich, St. Louis, MO, USA) with a lab blender Stomacher^®^ 80 (Seward, Worthing, UK) for 1 h, and 100 µL of serial dilutions 10^−3^ to 10^−7^ was plated on T4 agar plates (Ø 90 mm). Colonies developed on plates were counted after 48 h of incubation at 28 °C, and CFU g^−1^ was calculated by considering serial dilutions used (the trial was repeated three times in three independent experiments).

In post-emergence experiments, seedlings with the first leaves unfolded were inoculated after transplanting with a consortium solution of 10^6^ CFU mL^−1^ (adjusted spectrophotometrically by absorbance measurements at 600 nm). An amount of 10 mL of consortium solution was directly deposited to the base of each seedling.

In both experiments, infections were induced with FOC 10^6^ mL^−1^ spore suspensions (see Section 4.1). The experimental conditions investigated were (i) FOC, no bacterial inoculation/with fungal infection; (ii) Consortium + FOC, with bacterial inoculation/with fungal infection; (iii) Consortium, with bacterial inoculation/no fungal infection; and (iv) Control, no bacterial inoculation/no fungal infection. Each experimental unit consisted of 8 pots with 5 seeds/plants per pot (filled with 3 L of commercial common soil) left to grow in a greenhouse under a natural spring photoperiod (25–27 °C). Plants were checked daily and watered with 10 mL per plant every 2 days. The growth was stopped when infected plants showed evident disease symptoms 20 days after sowing for the pre-emergence trial and 30 days after sowing for the post-emergence trial.

Once growth was stopped, plants from both experiments were analyzed for the following parameters: germination/survival (%), plant height (cm), root length (cm), number of true leaves, total chlorophyll content (mg g FW^−1^) [45] and chlorophylls a/b ratio. The degree of damage was estimated as follows: 0 = no damages; 1 = 0.1–3 mm; 2 = 3–6 mm; 3 = 6–9 mm; 4 = 9–12 mm; 5 = > 12 mm/plant death.

### 4.9. Statistical Analysis

Mean values differences among experimental conditions were estimated by two-way analysis of variance (ANOVA). Comparison and separation of the means were performed by Fisher’s LSD post hoc test at a 5% level of significance (*p* < 0.05) using XLSTAT 2016 software (Addinsoft, Paris, France).

## Figures and Tables

**Table 1:** In vitro antagonistic activity of single bacterial strains and the bacterial consortium formed by *Burkholderia ambifaria*, *Gluconacetobacter diazotrophicus* and *Herbaspirillum seropedicae* against *Fusarium oxysporum* f. sp. *cannabis*.

Strains	Inhibition (%)
*Azospirillum brasilense*	<20%
*Burkholderia ambifaria*	65.0 a
*Gluconacetobacter diazotrophicus*	64.1 a
*Herbaspirillum seropedicae*	66.9 a
Consortium	70.6 a
LSD	6.7

The results are the mean of three replicates (three independent experiments). Results followed by the same case letter are not significantly different according to Fisher’s least significant difference (LSD) post hoc test (*p* < 0.05).

**Table 2:** In planta pre-emergence and post-emergence antagonistic activity of bacterial consortium formed by *Gluconacetobacter diazotrophicus*, *Herbaspirillum seropedicae* and *Burkholderia ambifaria* against *Fusarium oxysporum* f. sp. *cannabis*.

		Germination	C^g^	Damages	C^g^	Roots	C^g^	Shoots	C^g^	Leaves	C^g^	Chl tot	C^g^	Chl a/b Ratio	C^g^
Consortium	Pre	100 ^a^	^A^	-	^-^	3.3 ^c^	^A^	8.7 ^a^	^A^	4.3 ^c^	^A^	2.23 ^a^	^A^	4.99 ^d^	^AB^
	Post	100 ^a^		-		6.0 ^a^		6.5 ^c^		9.5 ^a^		0.49 ^d^		5.43 ^c^	
Control	Pre	100 ^a^	^A^	-	^-^	2.1 ^e^	^C^	4.0 ^e^	^C^	3.5 ^cd^	^B^	1.11 ^c^	^C^	3.26 ^f^	^B^
	Post	100 ^a^		-		3.6 ^c^		4.2 ^e^		5.5 ^b^		0.20 ^f^		6.70 ^a^	
Consortium + FOC	Pre	89 ^b^	^B^	2 ^c^	^B^	2.6 ^d^	^B^	7.6 ^b^	^B^	4.0 ^c^	^B^	1.78 ^b^	^B^	4.61 ^de^	^A^
	Post	85 ^c^		2 ^b^		5.2 ^b^		5.5 ^d^		6.0 ^b^		0.21 ^f^		6.12 ^b^	
FOC	Pre	55 ^d^	^C^	5 ^a^	^A^	1.4 ^f^	^D^	2.2 ^g^	^D^	1.5 ^e^	^C^	0.43 ^e^	^D^	1.02 ^g^	^C^
	Post	42 ^e^		5 ^a^		2.8 ^d^		3.0 ^f^		2.8 ^d^		0.02 ^g^		4.36 ^e^	
LSD Condition		1.4		0.3		0.2		0.4		0.6		0.04		0.31	
LSD Trial		0.9 *		0.2 *ns*		0.2 *		0.3 *		0.4 *		0.03 *		0.22 *	
LSD Condition x Trial		1.9		0.4		0.3		0.5		0.9		0.06		0.44	

In the Table: FOC, *Fusarium oxysporum* f. sp. *cannabis*; Pre, pre-emergence trial; Post, post-emergence trial; LSD, least significant difference; C^g^, Fisher’s LSD grouping based on Condition; Chl, chlorophylls; *, pre-emergence and post-emergence trials are significantly different based on Fisher’s LSD post hoc test; *ns*, pre-emergence and post-emergence trials are not significantly different based on Fisher’s LSD post hoc test. For the same column, results followed by the same case letter are not significantly different according to Fisher’s LSD post hoc test.

## Data Availability

The data that support the findings of this study are available upon request from the corresponding author.

## Supplementary Materials

The following are available online at https://www.mdpi.com/article/10.3390/plants10112436/s1, Table S1. Summary of multiple pairwise comparisons for Condition x Trial Interaction according to two-way ANOVA Fisher’s Least Significant Difference (LSD) post hoc test.
