# Supplementary material for: Fusarium Oxysporum f. sp. Cannabis Isolated from Cannabis Sativa L.: In Vitro and In Planta Biocontrol by a Plant Growth Promoting-Bacteria Consortium
Source: Plants (Basel). 2021 Nov 11;10(11):2436. doi: 10.3390/plants10112436 (PMC8623994; doi:10.3390/plants10112436)
Supplement: Supplementary file 1 [file plants-10-02436-s001.zip › plants-1389131-supplementary.pdf]

Supplementary material article

Fusarium oxysporum f. sp. *cannabis* isolated from *Cannabis sativa* L.: *in vitro* and *in planta* biocontrol by a plant growth promoting-bacteria consortium

Marika Pellegrini [1,*], Claudia Ercole [1], Carmelo Gianchino [1], Matteo Bernardi [1], Loretta Pace [1] and Maddalena Del Gallo [1]

[1] Department of Life, Health and Environmental Sciences, University of L'Aquila, Coppito 1, 67100 L'Aquila, Italy; marika.pellegrini@univaq.it (M.P.); claudia.ercole@univaq.it (C.E.); carmelo.gianchino@graduate.univaq.it (C.G.); matteo.bernardi1@graduate.univaq.it (M.B.); loretta.pace@univaq.it (L.P.); maddalena.delgallo@univaq.it (M.D.G)

* Correspondence: marika.pellegrini@univaq.it; Tel.: +39 0862433246

Table S1. Summary of multiple pairwise comparisons for Condition x Trial Interaction, according to two-way ANOVA Fisher’s Least Significant Difference (LSD) post-hoc test.

**Interaction**	Mean	Groups						
Germination %								
CONTROL*POST	100	A						
CONSORTIUM*POST	100	A						
CONTROL*PRE	100	A						
CONSORTIUM*PRE	100	A						
CONSORTIUM+FOC*PRE	89		B					
CONSORTIUM+FOC*POST	85			C				
FOC*PRE	55				D			
FOC*POST	42					E		
Damages grade (0-5)								
FOC*PRE	5	A						
FOC*POST	5	A						
CONSORTIUM+FOC*POST	2		B					
CONSORTIUM+FOC*PRE	2			C				
CONTROL*POST	0				D			
CONTROL*PRE	0				D			
CONSORTIUM*POST	0				D			
CONSORTIUM*PRE	0				D			
Roots lenght (cm)								
CONSORTIUM*POST	6	A						
CONSORTIUM+FOC*POST	5		B					
CONTROL*POST	4			C				
CONSORTIUM*PRE	3			C				
FOC*POST	3				D			
CONSORTIUM+FOC*PRE	3				D			
CONTROL*PRE	2					E		
FOC*PRE	1						F	
Shoots lenght (cm)								
CONSORTIUM*PRE	8,7	A						
CONSORTIUM+FOC*PRE	7,6		B					
CONSORTIUM*POST	6,5			C				
CONSORTIUM+FOC*POST	5,5				D			
CONTROL*POST	4,2					E		
CONTROL*PRE	4,0					E		
FOC*POST	3,0						F	
FOC*PRE	2,2							G

Table S1. *Cont.*

Leaves n°								
CONSORTIUM*POST	10	A						
CONSORTIUM+FOC*POST	6		B					
CONTROL*POST	6		B					
CONSORTIUM*PRE	4			C				
CONSORTIUM+FOC*PRE	4			C				
CONTROL*PRE	4			C	D			
FOC*POST	3				D			
FOC*PRE	2					E		
Total chlorophylls								
CONSORTIUM*PRE	2,23	A						
CONSORTIUM+FOC*PRE	1,78		B					
CONTROL*PRE	1,11			C				
CONSORTIUM*POST	0,49				D			
FOC*PRE	0,43					E		
CONSORTIUM+FOC*POST	0,21						F	
CONTROL*POST	0,20						F	
FOC*POST	0,02							G
Chlorophylls a/b ratio								
CONTROL*POST	6,7	A						
CONSORTIUM+FOC*POST	6,1		B					
CONSORTIUM*POST	5,4			C				
CONSORTIUM*PRE	5,0				D			
CONSORTIUM+FOC*PRE	4,6				D	E		
FOC*POST	4,4					E		
CONTROL*PRE	3,3						F	
FOC*PRE	1,0							G
